# Editorial: Bidirectional gut-brain interactions in modulating central nervous system diseases

**DOI:** 10.3389/fcell.2026.1924917

**Published:** 2026-09-09

**Authors:** Yan Gao, Yushu Ma, Liana Asatryan, Dalia Ashour, Yiming Meng

**Affiliations:** 1 Department of Neurology, Shengjing Hospital of China Medical University, Shenyang, Liaoning, China; 2 Department of Central Laboratory, Cancer Hospital of Dalian University of Technology, Liaoning Cancer Hospital & Institute, Shenyang, Liaoning, China; 3 Titus Family Department of Clinical Pharmacy, Mann School of Pharmacy and Pharmaceutical Sciences, University of Southern California, Los Angeles, CA, United States; 4 Medical Parasitology Department, Faculty of Medicine, Tanta University, Tanta, Egypt

**Keywords:** central nervous system diseases, dysbiosis, gut-brain interactions, microbiome, neuroinflammation

The relationship between the gastrointestinal tract and the central nervous system (CNS) has undergone a profound conceptual evolution over the past 2 decades. Once considered a largely unidirectional axis in which the brain governed gut function, this relationship is now recognized as a sophisticated, bidirectional communication network integrating neural, endocrine, immune, and microbial signals. The gut microbiota-comprising trillions of bacteria, fungi, viruses, and archaea-encodes a metabolic repertoire that rivals the human genome in complexity, producing neuroactive metabolites, modulating systemic immune tone, and influencing blood-brain barrier integrity. Simultaneously, CNS perturbations ranging from acute injury to chronic psychiatric illness reshape microbial composition, intestinal motility, and epithelial barrier function. This reciprocal vulnerability has catalyzed enormous research interest, not only in understanding disease mechanisms but in identifying the gut as an accessible and modifiable therapeutic target.

The ten contributions assembled here span cerebrovascular, neuropsychiatric, neuro-oncological, iatrogenic, and extra-cranial disease contexts. Collectively, they delineate four converging themes: (i) microbial dysbiosis as both a consequence and a driver of CNS pathology; (ii) the centrality of barrier integrity in shaping disease trajectory; (iii) microbiota-derived metabolites as *bona fide* neuroactive molecules; and (iv) the still-incomplete translation of these insights into standardized therapeutics.

Stroke represents one of the most intensively studied contexts for gut-brain interaction, and two contributions in this Topic address complementary dimensions of this relationship. Jia et al. reported that the large intestine functions not merely as a digestive organ but as a critical immunological and neuroendocrine interface in ischemic stroke, detailing how intestinal epithelial cells, barrier integrity, and the enteric neural network are disrupted by cerebrovascular injury while simultaneously feeding back to modulate neuroinflammatory cascades in the ischemic brain (Jia et al.). Extending this mechanistic foundation to therapeutic translation, Wang et al. systematically reviewed gut microbiota-based strategies for stroke prevention and treatment, evaluating the evidence for probiotics, prebiotics, fecal microbiota transplantation (FMT), and dietary interventions, while underscoring persistent challenges including individual microbiome heterogeneity, efficacy stability, and long-term safety (Wang et al.). A particularly important neuropsychiatric sequela of stroke is addressed by Jiang et al., who proposed a novel microbiota-immune-barrier axis to explain post-stroke depression (PSD) (Jiang et al.). Their review argues that ischemic stroke-induced gut dysbiosis compromises both intestinal and blood-brain barrier integrity through dysregulated immune signaling, thereby precipitating or amplifying depressive symptoms in more than one-third of ischemic stroke survivors - a population that remains substantially undertreated in clinical practice.

Spinal cord injury (SCI) presents a distinct but mechanistically related challenge. Yin et al. provided a comprehensive bench-to-bedside synthesis of the emerging “gut microbiota-gut-spinal cord” axis, demonstrating the bidirectional relationship between SCI and gut microbiota SCI disrupts autonomic control of intestinal function and triggers gut dysbiosis, while microbial metabolites, notably short-chain fatty acids (SCFAs) and aryl hydrocarbon receptor (AhR) agonists, traverse the blood-spinal cord barrier to exacerbate secondary neuroinflammation. Meanwhile, gut microbiota and its metabolites can modulate SCI, reduce neuroinflammation and promote neural repair. Critically, their review positions gut microbiota as both prognostic biomarkers of injury severity and promising therapeutic targets, while acknowledging that standardized, clinically validated microbiome-based interventions for SCI remain an unmet need.

Psychiatric and functional gastrointestinal disorders are represented by two mechanistically rich contributions. Lambiase et al. examined the role of GABAergic dysregulation in irritable bowel syndrome (IBS), demonstrating that GABA receptors and transporters operate at multiple levels-CNS circuits, the enteric nervous system, and gut epithelium-and that gut microbiota-derived GABA production directly links microbial composition to inhibitory neurotransmitter tone within the microbiota-gut-brain axis (Lambiase et al.). Altered GABA signaling and reduced GABA levels, particularly in diarrhea-predominant IBS subtypes, suggest that GABAergic pathways represent both a disease mechanism and a therapeutic target, though the authors note that clinical evidence for GABA-based interventions remains limited and largely preclinical. Addressing the comorbidity of depression and constipation through a translational lens, Xu et al. reported that the traditional Chinese medicine formula Liqi Yangyin (LQYY) ameliorates chronic unpredictable mild stress (CUMS)-induced behavioral changes and intestinal dysmotility through ACE/FFAR2-mediated modulation of the microbiota-gut-brain axis, with downstream effects including reduced microglial neuroinflammation, restored intestinal and blood-brain barrier integrity, and enhanced colonic serotonin secretion-mechanistic findings subsequently confirmed in BV-2 cell culture models (Xu et al.).

Expanding beyond classical gut-brain dyads, several contributions introduce novel axis frameworks with broad clinical implications. Zhang et al. explored the interaction between gut microbiota and anesthesia through the lens of the gut-brain-liver axis, revealing that anesthetic agents reshape microbial composition by disrupting intestinal barrier function, suppressing beneficial bacterial populations, and inducing bile acid metabolism dysregulation-changes that in turn impair hepatic drug-metabolizing enzyme activity and sustain neuroinflammatory cycles with direct perioperative safety consequences (Zhang et al.). Song et al. proposed a brain-gut-prostate axis in chronic prostatitis/chronic pelvic pain syndrome (CP/CPPS), integrating microbiome-derived metabolite signaling (including SCFAs and thyrotropin-releasing hormone), neuroendocrine pathways, and Th17/Treg immune imbalance to construct a theoretical framework that transcends conventional localized anti-inflammatory approaches and offers multi-dimensional therapeutic strategies for this clinically refractory condition (Song et al.). Zhai et al. examined Drug-induced brain injury (DIBI) through the gut-brain axis, demonstrating that antibiotic, antineoplastic, and psychoactive drug exposures promote microbial dysbiosis, bacterial translocation, and systemic inflammation that ultimately compromise blood-brain barrier integrity and drive oxidative stress, neuroinflammation, and metabolic dysfunction-highlighting that iatrogenic gut disruption is itself an underappreciated pathway to CNS harm (Zhai et al.). Finally, Kang et al. provided the first systematic metagenomic and metabolomic characterization of gut microbiota in primary CNS lymphoma (PCNSL), reporting elevated Proteobacteria abundance, a raised Firmicutes/Bacteroidetes ratio, reduced oleamide, and elevated deoxycholic acid in patients relative to healthy controls, with KEGG pathway enrichment implicating nitrogen, phenylalanine, purine, and pyrimidine metabolism—findings that open a new avenue of inquiry into microbiome contributions to CNS oncogenesis (Kang et al.).

Across these ten contributions, three priorities emerge for the next phase of the field. First, mechanistic specificity must be sharpened: SCFA-FFAR2, AhR-tryptophan, bile acid-FXR, and GABAergic axes should be tested in disease-specific contexts rather than invoked as generic explanations. Second, barrier biology deserves equal billing with microbial composition: several articles in this Topic converge on the view that compositional dysbiosis is pathogenic only insofar as it compromises intestinal, blood-brain, or blood-spinal cord barriers. Third, and most pressingly, clinical translation remains the bottleneck. Inter-individual heterogeneity, the absence of standardized endpoints, and uncertainty regarding long-term safety-candidly acknowledged by Wang et al., Zhang et al., and Yin et al.-continue to limit the migration of microbiota-targeted therapies from the bench to routine practice. Multi-omics integration, well-powered randomized trials, and precision-stratified patient selection are now indispensable.

